# Innovative Plant-Based Burger Enriched with *Tenebrio molitor* Meal: Characterization and Shelf-Life

**DOI:** 10.3390/foods12183460

**Published:** 2023-09-16

**Authors:** Paula Ardila, Adrián Honrado, Pedro Marquina, José Antonio Beltrán, Juan B. Calanche

**Affiliations:** Instituto Agroalimentario de Aragón–IA2-(Universidad de Zaragoza-CITA), Miguel Servet 177, 50013 Zaragoza, Spain; 865437@unizar.es (P.A.); adrihonfri@unizar.es (A.H.); pmarquina@unizar.es (P.M.); jbeltran@unizar.es (J.A.B.)

**Keywords:** *T. molitor* flour, burger, shelf-life, based-to-plant, sensory analyses

## Abstract

Environmental concerns, among other causes, are leading to meat replacement in the diet by healthy, nutritious, and tasty foods. Alternative protein sources of plant origin can be an alternative to meat but their low biological value proteins can be a problem. Novel foods, such as insect meals, can meet current consumer’s demands. Therefore, this research has developed innovative prototypes of analog burgers with insect and vegetable proteins. Concerned about health and allergies, a prototype incorporating soya to satisfy coeliacs was developed. An iterative and heuristic process was carried out to test the product development feasibility. The main raw materials used were insect flour (*Tenebrio molitor*), seitan, and soya. In addition, oat and sodium alginate were used as binders. The shelf-life of the new product was evaluated by physicochemical (pH, a_w_, moisture, color, acidity, and peroxide index) and sensory analysis (quantitative analysis QDA). The production of the burger analogs was feasible. Product characterization showed significant differences (*p* < 0.05) among samples for organoleptic properties, highlighting texture changes. Using a multivariate model, it was established that the “best before date” occurs at seven days for all developed prototypes, conditioned by microbial growth. Finally, the spoilage model indicated an important contribution to bacterial growth with a notable modification to the pastiness and hardness of the burger analogs developed.

## 1. Introduction

Meat consumption is increasing and is expected to continue growing. To meet this demand, industrial livestock production is multiplying and debates about the environmental, ethical, and health consequences of large-scale meat production are raising. The environmental impact is reflected in increased greenhouse gas emissions, water pollution, and deforestation. For this reason, protein alternatives, like those coming from plants, are being sought, trying to simulate the texture and taste of meat [1]. The term meat analogs distinguishes food products that are not made from meat. Since 2015, the supply of meat substitute products has increased. That is why new strategies are being considered to cause a change in consumer expectations, including the development of new products such as burgers, nuggets, and other meat products [2].

Meat substitutes could be a vehicle for increasing the consumption of cereals and legumes as most of the meat analogs that are successfully marketed are derived from plant proteins [3]. These foods are perceived as healthy and sustainable, in contrast to meat. The most recognized by consumers are tofu, seitan, and textured soya. They are fibrous vegetable proteins that are cholesterol-free, low in fat, high in protein and fiber, and rich in other nutrients, depending on the type of vegetable. Proteins are usually extracted from plants such as soya, peas, or beans and can be used in different forms: isolated, concentrated, or as flour. They can be mixed with ingredients to provide different textures and mouthfeel [1]. Soya has a high nutritional value and is a rich source of protein and bioactive phytochemicals, such as gamma-tocopherols and isoflavones [4]. Textured soya protein is obtained from defatted soya flour from which soluble carbohydrates have been removed. The filtrate obtained is textured using extrusion technologies. This methodology provides a specific texture: expanded, molded, or textured, like meat. In addition, it possesses functional properties such as water retention capacity, gelling power, fat absorption, and emulsifying capacity. Soya is also a gluten-free cereal suitable for the coeliac diet [5]. Another alternative is seitan, a meat alternative produced from wheat flour gluten, a protein with excellent chewiness and taste properties. It is a product with popular tradition in China [6]. It is produced by removing starch from wheat flour dough by washing, resulting in a chewy gluten dough [7]. Furthermore, it is responsible for the formation of meat-like structures, water retention, and stabilization during formulation [6]. Food products containing wheat gluten work as meat extenders and meat analogs. Meat substitutes incorporating seitan are more economical because wheat cultivation is widely distributed throughout the world. On the other hand, oat is a popular cereal because it affects the glycemic index and gut microbiota control. It has a high content of quality protein determined by amino acids (lysine, asparagine, aspartic acid, and alanine). The main properties of oat include the fat-binding capacity and moisturizing, emulsifying, foaming, and gelling properties, which can be a positive point for innovation in new foods [1]. Oat protein is being studied as a possible functional ingredient in the incorporation of new foods [8]. In addition, oat has antioxidant compounds that can keep oils and fats stable against rancidity.

Meat substitutes have advantages because many plant-origin foods can be combined or used. Another important fact to consider is that these foods can be made for people with dietary diseases, such as those with gluten intolerances. One percent of the world’s population has coeliac disease. Coeliac disease is a chronic disease that affects people, causing them to be unable to consume foods containing gluten. These people often suffer from disorders such as difficulty absorbing nutrients from food, diarrhea, abdominal pain, vomiting, and weight loss. In this sense, the food industry is increasing the development of foods for coeliacs due to their limited variability, so the use of gluten-free cereals such as those mentioned above could be an interesting option [9].

However, meat analogs have several drawbacks. The main difficulty is people’s low inclination to switch from animal-based to plant-based foods in their diet [10], in addition to the technological and sensory development difficulties involved in producing these foods. Given that meat is an essential food in the diet, an analog with the organoleptic characteristics of meat is difficult to reproduce. This makes their success limited. However, the new generation of meat analogs is becoming increasingly palatable due to the modification of their textural characteristics, color, and flavor enhancement, using natural colors and flavorings [3]. However, the main drawback of the meat analogs is the low biological quality of plant-based protein [11].

At the same time, the consumption of protein sources from unconventional raw materials, such as insects, is becoming an option to improve the nutritional value of novel foods as it is an easy and convenient way to enrich foods by means of alternative protein sources [12]. Alternative insect protein sources symbolize a new milestone in people’s diets, but food neophobia, unfamiliarity, and poor sensory attributes have been identified as the main barriers preventing people from choosing alternatives to meat [11]. The consumption of insects is supported by the term entomophagy, an ancient custom practiced in many countries around the world, but especially in Southeast Asia, Africa, and Central and South America. In contrast, in Western countries, insect consumption is lower due to the cited neophobia [13]. Insects are an alternative source of animal protein that currently is being studied due to their economic, environmental, and sustainable benefits. Among the most consumed insects is the yellow mealworm (*Tenebrio molitor*) as they become pests in stored foodstuffs such as flour and cereals. This is because their main habitat is characterized by dark and damp places [14]. The production of these insects is intended to be incorporated into human food as the larvae are more acceptable to European consumers. They can be marketed as whole or in flour form to facilitate their incorporation into food products. Insects are included in the definition of “novel food” [15]. Larvae are a good source of nutrients, rich in essential amino acid-based, high-value protein, vitamins (vitamin E, vitamin B12, niacin, riboflavin, pantothenic acid, and biotin), and minerals (P, Mg, Zn, and Mn) [14]. The FAO classifies insect consumption within the Sustainable Development Goals (SDGs).

The stimulus of the development of these novel foods is expected to continue increasing. Therefore, this research aimed to investigate the feasibility of using unconventional plant-based raw materials for the development of burger alternatives, specifically one using seitan for the general population and another with soy for individuals with celiac disease. Additionally, to enhance the nutritional profile of these products, *T. molitor* meal was incorporated. To achieve these objectives, a heuristic and iterative approach to formulate the final recipes was employed. Simultaneously, a comprehensive shelf-life assessment was conducted, evaluating physicochemical, microbiological, and sensory parameters, allowing the establishment of an optimal “best-before” date.

## 2. Materials and Methods

### 2.1. Raw Material

The main raw material of vegetable origin in the developed burger analogs consisted of seitan, textured soya (suitable for coeliacs), and oat. They were purchased from a Spanish supermarket (Mercadona, Tabernes Blanques, Spain). Seitan was manufactured by Midsona Iberia S.L.U (Castellterçol, Spain); textured soya was from Laboratorios Almond, S.L (Librilla, Spain), and the oats were purchased in flakes (Brüggen^®^, Lübeck, Gemany) and ground in the laboratory with a mincer (Mod. A320R1, Moulinex^®^, Alençon, France). 

Insect (*T. molitor*) larvae flour, an alternative protein source, was used to achieve the enrichment of the developed products. It was supplied by the company Insekt Label Biotech S.L. (Bizkaia, Spain). In addition to these products, sodium alginate (Solegraells^®^, Barcelona, Spain), calcium chloride (Carlo Erba^®^, Sabadell, Spain), aromas and species varieties (Carinsa S.L, Barcelona, Spain), oat flour (Brüggen^®^, Lübeck, Gemany), 100% vegetable spreadable fat (Mercadona, Tabernes Blanques, Spain), beetroot powder coloring (Sosa Ingredients S.L, Navarcles, Spain), sweet paprika powder (Mercadona, Tabernes Blanques, Spain), and salt (Alcampo, Madrid, Spain) were used as ingredients for the burger analogs.

### 2.2. Experimental Design

**Figure 1:**
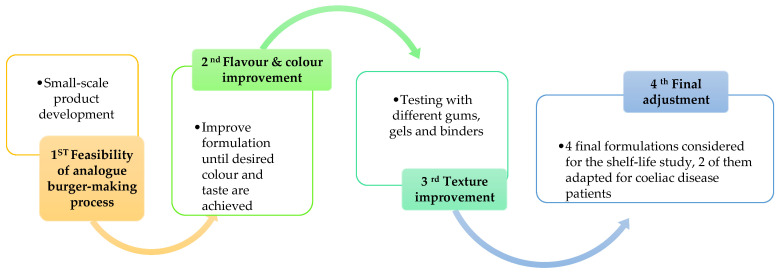
Burger analogs-making iterative and heuristic process.

The procedure carried out to develop the burgers was a heuristic methodology based on an iterative process [16]. The results of the process were the starting point for the following tests until a product that met the predefined requirements was attained. Therefore, a test was previously carried out to determine the feasibility of the development of these new products. Once these expectations were met, improvement in the formulation was carried out until an acceptable taste was reached. Then, the texture was worked on. Texture implies a challenge in the production of a meat product from vegetable proteins. Figure 1. shows how the iterative process was developed until 4 final formulations were reached [17].

### 2.3. Burger Analog Production

Twenty-five different formulations were elaborated in the iterative process until the four final formulations were reached. A mixer grinder was used to make the burger (Robot Coupe, Mod. Blixer 6 V.V) and Petri dishes were used to give their characteristic shape, with an exact weight of 80 g per unit. The final formulations are shown in Table 1. The samples were packed in air-filled trays using a thermosetting machine (Tecnotrip, Mod. EV-13). Once packaged, the samples were stored at 4 °C until analysis.

### 2.4. Physicochemical Quality of Developed Products

#### 2.4.1. pH

The pH was measured with a digital puncture pH meter (XS instruments, Mod. PH25) previously calibrated at pH 7 and pH 4 following device instructions. The pH meter was inserted into the sample and then, researchers waited until the reading was stable, according to the manufacturer’s instructions.

#### 2.4.2. Color

The color parameters were evaluated using a colorimeter (Minolta CO, Mod. CM-2002) to determine C.I.E Lab using the coordinates L* (lightness), a* (red/green coordinates), and b* (yellow/blue coordinates). The color of these products was measured raw from the four formulated samples.

#### 2.4.3. Moisture (H%)

The moisture was measured for each sample, for the analysis, according to the gravimetric method employing a thermobalance (KERN and Sohn GmbH ^®^, Mod. DBS 60-3). 

#### 2.4.4. Water Activity (a_w_)

This parameter was evaluated for each burger analog sample using automatic measuring equipment of a water activity meter (Decagon Devices, Mod.CX-1) using the protocol described in the user’s manual. A small sample was taken and the samples were placed in the containers of the equipment and measured.

#### 2.4.5. Acidity Index

The acidity index was determined according to COPANT, 1969 [18]. To a 5 g of sample, 25 mL of neutralized ethanol was added and then homogenized with an Ultra-Turrax (IKA, Staufen, Germany). Afterwards, samples were centrifuged, filtrated, and titrated with a solution of NaOH 0.1 N. The acidity index is expressed in % oleic acid/100 g.

#### 2.4.6. Peroxide Index

The peroxide value was determined according to ISO 3960:2017 (Modified) [19]. Then, 10 g of the sample were weighed and 25 mL of n-hexane were added. The sample was homogenized, centrifuged, and filtrated into a 100 mL Erlenmeyer flask (Duran, Sabadell, Barcelona). In total, 5 mL of the supernatant was pipetted and 7.5 mL of glacial acetic acid (Carlo Ebra, Sabadell, Spain) and 0.2 mL saturated KI (PanReac, Ottoweg, Germany) were then added. To improve the mixing of the reagents, it was stirred for 2 min. After this time, 25 mL distilled water was added and 1% starch (PanReac, Ottoweg, Germany) was used as an indicator. The sample was stained greyish and titrated with sodium thiosulphate 0.002 N. Peroxides values were expressed as meq O_2_/Kg.

### 2.5. Microbiological Quality of Burger Analogs

The study of microbial growth allowed the development of a spoilage model. Total viable counts (TVC) at 37 °C were conducted according to ISO 4833-1:2013 [20]. Furthermore, a count of molds and yeasts (MY) was made following ISO 21527-1:2008 [21]. For the enumeration of the *Enterobacteriaceae* family bacteria (ET), the standard ISO 21528-2:2017 [22] was followed. At each of the sampling points (days 1, 5, 8, and 12), 10 g of each fresh burger was taken and placed in a sterile plastic bag with 90 mL of peptone water (0.1%). After 2 min in a stomacher blender (Mod. 1986/470, IUL Instruments, Spain), appropriate decimal dilutions were poured and plated (1 mL) on the following media: Plate Count Agar (PCA) for the total viable count (37 °C for 48 h and 10 °C for 5 days, respectively) and Violet Red Bile Glucose Agar (VRBG) for *Enterobacteriaceae* (37 °C for 24 h). On the other hand, the sabouraud agar medium supplemented with chloramphenicol was used for mold and yeast counts. All microbial counts were converted to logarithms of colony-forming units per gram (log CFU/g). 

### 2.6. Sensory Study of Burger Analogs

The different sensory analyses carried out had two main purposes: first, establish the general sensory profiles of the new products. Second, evaluate their acceptance by consumers. Samples for sensory analysis were cooked in a frying pan with olive oil for two minutes on each side until a core temperature of 75 °C was reached; the temperature was controlled with a puncture probe. The samples were served to the participants in equal parts (20 g) at 45 °C. All ingredients used in the study are safe for human consumption and sensory evaluators were informed of samples’ composition. Therefore, there was no need for an ethical committee’s approval for the experiment. 

#### Quantitative Descriptive Analysis (QDA)

A trained panel of 10 assessors [23,24] was used according to the QDA methodology. The analytical sensory evaluation was developed according to the procedure of Gacula (1997) for the sensory characterization of burger analogs [25]. Structured scales anchored at the extremes (0, attribute not present −10, attribute very present) were used for each of the specific sensory attributes highlighted in the penalty analyses (ISO 13299:2016) [26]. The analyses were carried out using SENSESBIT^®^ software. For the selection of the sensory attributes to evaluate the hamburger analogs, the flash profiling technique based on the free choice of attributes was used [27]. The selected descriptors were the homogeneity and color intensity (similarity to beef burger) for visual assessment; vegetable aroma, cooked meat aroma, smoked aroma, spices aroma, and rancid odor for olfactive assessment; elasticity, firmness, fracturability, crunchiness (at first bite), hardness, succulence, cohesiveness, graininess, pastiness, and gumminess (while chewing) for texture assessment; and umami taste, salty taste, bitter taste, cooked meat taste, vegetable taste, and aftertaste persistence for taste assessment [27]. The sessions were held in the tasting room of the Pilot Plant of the Faculty of Veterinary. In these tastings, the burger analogs were characterized from the starting point of the experiment, according to UNE-EN-ISO 8586:2014 [23]. 

### 2.7. Shelf-Life Study for Burger Analogs Developed

#### 2.7.1. Shelf-Life Design

A shelf-life study was developed considering the recommendations established in ISO 16779: 2015 [28]. An experimental design was performed following a basic overall design with a statistical approach [29]. A Drawn Simple Design (DSD) model was used to describe the evolution of the prototypes over time under the same storage conditions (4 °C). A total of 4 sampling points were performed on day 1, 3, 8, and 12. The study was carried out until typical sensory defects were detected in the product. A database was compiled with the results obtained for the physicochemical and microbiological parameters studied. On each sampling day, the same parameters used for characterization were analyzed: physicochemical, microbiological, and sensory analyses.

#### 2.7.2. Shelf-Life Model (Multivariate Method)

According to several authors [16,30], the shelf-life according to a multivariate criterion considers the totality of the physicochemical and microbiological analyses performed on the sample developed to estimate the shelf-life of the burger [29]. These results were statistically analyzed by Analysis of Variance (ANOVA) and Principal Component Analysis (PCA) tests to estimate the shelf-life according to multivariate criteria. Using the factor scores obtained in the first component (F1) on each of the sampling days, a scatter plot was later constructed where the cut-off points with the abscissae represented the ideal shelf-life of the product.

#### 2.7.3. Spoilage Descriptive Model

This model was carried out with the results of the parameters analyzed in order to understand how they acted during its storage. For this purpose, a least squares regression statistical analysis (PLS) was supported. This allowed us to understand the relationships among variables (physicochemical, microbiological, and sensory parameters) for the developed products.

### 2.8. Statistical Analysis

The measurements of the analyses were carried out in triplicate. The results obtained from the different physicochemical, microbiological, and sensory analyses accomplished for this work were analyzed by descriptive and inferential statistics using Microsoft Excel with XLSTAT software (Addinsof^®^, version 16). A univariate analysis was previously performed on the data set, with the calculation of maxima, minima, quartiles, means, modes, and variances, represented graphically by box plots, scattergrams, and histograms, to check the normality of the data and to detect non-parametric values. Bivariate comparisons were held by proving Pearson’s correlation coefficients. An Analysis of Variance (ANOVA) was then carried out to establish the differences between the different variables studied. For this purpose, a 95% confidence interval was considered and significant differences were considered when *p* < 0.05. Principal Component Analysis (PCA) was used as an exploratory and graphical method for the shelf-life study. In the sensory characterization of the developed products, an ANOVA was previously tested. A panel analysis was then performed to ensure the consistency of the judges’ evaluations. Subsequently, product characterization was analyzed using the cosine squared method (Cos^2^) and corrected means were estimated.

## 3. Results and Discussion

### 3.1. Iterative Development Process

The procedure followed to obtain the final formulations began with a study of the basic formulations used on the market to make hamburgers. From there, the feasibility of using raw materials such as seitan was studied. On the other hand, a gluten-free raw material, in this case, soya, was chosen for the coeliac population. Product development was feasible and a soy-based hamburger was produced. All these experiments were carried out on a small scale. This milestone resulted in several authors confirming that a plant-based hamburger prototype development was possible [31]. The next point to work on was to correct the taste. The taste was modified and improved by using flavorings and spices, masking agents reminiscent of a traditional beef burger. The taste was satisfactorily achieved and the use of insect flour added umami and cereal flavors. On the other hand, among the main challenges encountered when designing the formulation was the search for a meat-like texture. As some authors claim, it is a challenge to find a texture equal to that of a beef burger [32]. Information about binders was found in the literature [33]. Some binders were used to provide a consistent texture. Gellan gum, xanthan gum, guar gum, and a mixture of sodium alginate with calcium chloride were tried but they provided an undesirable mouthfeel which masked the specific flavors of the burgers. Finally, the only gel that did not mask the typical flavors was sodium alginate in combination with calcium chloride, which provided a texture very similar to that of a beef burger. However, in order to achieve a clean label that would be more appealing to the consumer, the use of natural ingredients was pursued, in this case wheat flour, rice, oats, white corn, and maize starch. Depending on the type of flour used, these provided a strong vegetable flavor, as in the case with chickpea flour, evocative of falafel. However, tests with oat flour were able to provide a very pleasant and toasted taste. After 25 prototypes, 4 final formulations for burger analogs were achieved: seitan + alginate (SEAL), seitan + oat (SEOA), soya + alginate (SOAL), and soya + oat (SOOA) (Table 1).

### 3.2. Quality and Safety of Burger Analogs

The burger analogs formulated with alginate obtained higher AI values (*p* < 0.001) compared to those made with oat. The above could be by the hydrolysis of acyl glycerides, mainly due to lipase enzyme activity. In the scientific literature, oat is studied for their antioxidant activity. Furthermore, in these samples with alginate, the peroxide index was significant (*p* < 0.001), correlating with the AI results. Alginate-based formulations for meat analogs have been reported as highly vulnerable to oxidative lipids reactions [33]. The pH, in general, was around 6.4 for most of the samples. The pH is at these values is due to the slightly alkaline character of the ingredients used in the formulations. The values obtained agree with those obtained by De Marchi et al. (2021), who obtained a pH of 5.58−7.9 for a burger of vegetable origin [34]. According to Botella-Martínez et al. (2022), pH has a relevant effect on the final color as vegetable ingredients can change color depending on the pH of the food [35]. Water activity (a_w_) did not show relevant differences between samples and characteristics of meat preparations, which makes it a product in which microorganisms could grow and proliferate, leading to alterations in the product. In terms of moisture, values between 49.27−58.81%, were obtained, characteristic of the product prepared. The color was characteristic of the hamburger analogs because the color was achieved by natural coloring, trying to imitate raw meat. The values of the different coordinates were found to be within the range: *L (36.74–44.29), a* (8.73–9.95), and b* (16.44–19.72). These values were mainly due to the difference in raw material. Seitan has a brownish color while soya is usually pale yellow. However, coloring agents were used in order to simulate the red color of meat. The measurements were carried out raw because after cooking, the color changed to brownish due to Maillard reactions. The different values were obtained for the different coordinates. 

The SEAL sample had a lower luminosity compared to the other products. This may be because seitan in this case has a darker coloring than soya. The a* coordinate could differentiate the products according to the main raw material (*p* > 0.01). Formulations with soya have a higher reddish color compared to those formulated with seitan, which may be because the dye was more potent when introduced into a light color matrix. For the b* coordinate, the SOOA formulation was the least yellow colored (*p* > 0.05). The other samples obtained very similar results, as can be seen in Table 2, indicating a yellower color than the other burger analogs. In the study by De Marchi et al. (2021), the color analysis for meat and vegetable burgers yielded very similar values to those found in this study; obtaining a value of L* between 42.36–48.61 and 39.87–48.90 for meat and vegetable-based burgers, respectively. It was also observed that the b* values were closer to the meat-based burger analog of 13.57–15.88 while the a* values were different. This could be because the samples analyzed in the study by De Marchi et al. (2021) had a higher number of ingredients that could better simulate the real color of meat preparation [34].

One of the objectives of this study was to fortify burger analogs by using *T. molitor* flour. In this sense, the developed products on average had a weight of 80 g and were fortified with 5.8 g of insect flour which provided approximately 3.5% of high biological value protein, promoting an increase in both its quantity and quality. As seen in the literature, the content of the burgers would be in the range of 18.6–19.4 g protein/100 g. This nutritional strategy is expected to outperform commercial plant-based burgers currently on the market [34,35].

### 3.3. Sensory Characterization of the Burger Analogs

**Figure 2:**
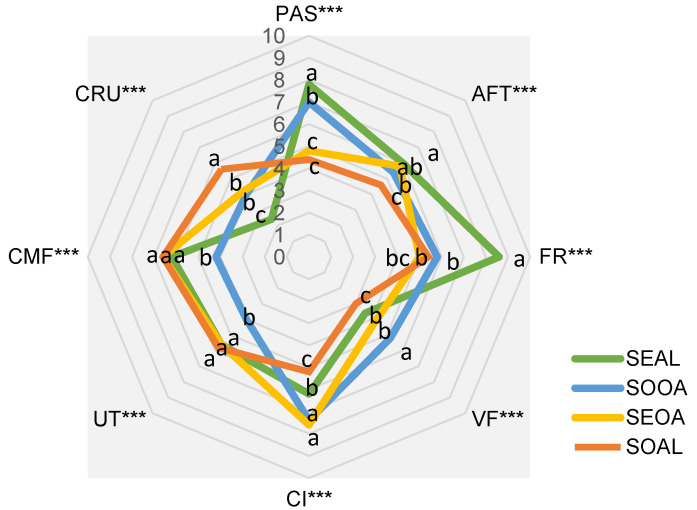
Development of the initial product characterization (spider diagram) on day 1. Shows the characterization of the product as a function of the adjusted mean representation, where the attributes characterizing the treatments are represented, as mentioned above. CRU: Crunchiness; CI: Color Intensity; FR: Fracturability; PAS: Pastiness; VF: Vegetable flavor; AFT: Aftertaste UT: Umami taste; HAR: Hardness; CMF: Cooked meat flavor. Different letters indicate significant differences, *** (*p* < 0.001).

This sensory analysis demonstrated the discriminatory power of each attribute, as can be seen in Figure 2.

The following attributes stood out as highly significant (*p* < 0.001) and therefore with the highest discriminatory power: crunchiness, rancid odor, cooked meat aroma, color intensity, fracturability, pastiness, vegetable flavor, aftertaste persistence, and umami taste (Figure 2). This spider graph shows the initial time formulations with the eight most significant attributes for the formulations studied. The rancid odor was removed from the graph because it is considered a negative attribute in this case to characterize these products. The SEAL burger was characterized by the most pastiness and fracturability texture; the crunchiness in this formulation was less noticeable. This may be because the alginate together with the seitan resulted in a softer texture, not forming a good cohesion of the ingredients. In the taste dimension, the umami taste was like the other burgers. In relation to appearance, it was different from the others. This formulation had the longest persistence of aftertaste. The SOOA formulation remained near to the SEAL formulation in texture, as it was perceived as pasty and fracturable to a lesser extent, but these traits characterized it. On the other hand, it was the one with the most vegetable flavor. As the cooked meat flavor decreased, this may be because soya and oat have a prominent vegetal aroma, which covered the added flavors. It presented itself as the burger with the highest color intensity. The umami taste was absent, which is reflected in the lack of persistence in the aftertaste. The SEOA burger maintained a texture closer to that of a burger, as it was not perceived as too pasty and was not so fracturable. In appearance, it stood out as the one with the highest color intensity as well as its oat counterpart, this may be because the oat-based formulations were more toasted during cooking. In the flavor and aroma dimension, it was perceived with a pronounced umami flavor, cooked meat flavor, and a minor level of vegetable flavor. In addition, it retained an aftertaste. The crunchy character was perceived to be the same as in the oat burger. Oat, therefore, characterized the crispiness and color equally in the formulations. Finally, the SOAL was the least pasty and fracturable burger of all the formulations and stood out as the crunchiest of all. As far as flavor is concerned, it stood out for it is cooked meat flavor, while the vegetable flavor was not so noticeable. The color was the least intense, which may be because the soya is not roasted as much during cooking. The umami flavor was prominent but the aftertaste was not as persistent.

**Figure 3:**
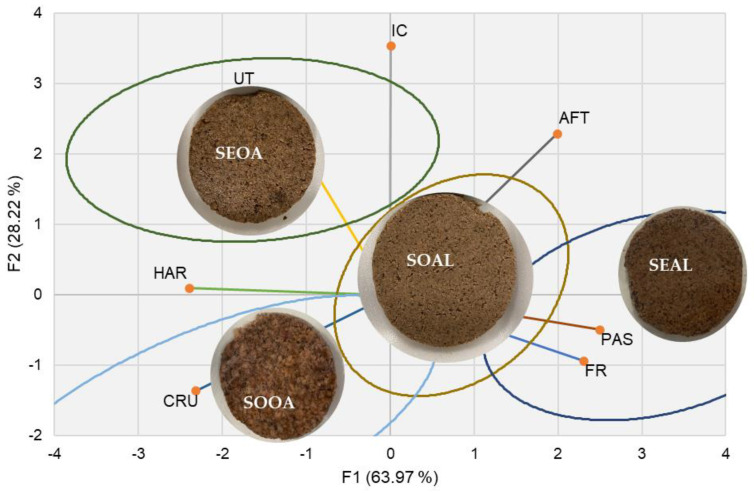
Sensory profiles based on Cos^2^ methodology for the developed products (burger analogs). CRU: Crunchiness; IC: Color Intensity; FR: Fracturability; PAS: Pastiness; VF: Vegetable flavor; AFT: Aftertaste UT: Umami taste; HAR: Hardness. SEOA: Burger analog seitan + oat; SOOA: Burger analog texturized soya + oat; SEAL: Burger analog seitan + alginate; SOAL: Burger analog texturized soya + alginate.

Finally, Figure 3 shows which sensory attribute defined each developed formulation. The SEOA burger stood out for it is umami taste, toughness, and color intensity and was near to the SOOA burger in terms of crunchiness and toughness. The SEAL burger was perceived to be more pasty and fracturable. And as a more neutral evaluation without being characterized by any attribute in general, the SOAL burger was found to be more neutral. Burgers with oats were found to be nearer to each other than those formulated with alginate; nevertheless, the raw material used. Oat is an ingredient that provides more hardness and gives a more intense color to the food.

The burgers with alginate showed differences between the burgers formulated with oat. In the study by Caparrós et al. [36] where a sensory analysis was carried out for four different types of burgers, including a hybrid of meat with insect flour and another of insect flours with lentils [36], it was observed that consumers, in both products, were able to identify the remains of the insect exoskeleton, which in most cases was reported as undesirable (defect) [37]. Similarly, in the present investigation, this ingredient (insect flour) was responsible for the crunchiness perceived by the trained sensory evaluators. It should be noted, however, that the granulometry of the insect flour used was very fine, making it visually very difficult to detect in the final products, although, as mentioned, for example, with SOOA, it was able to produce a considerable sensory stimulus. In the end, the sensory profile of the developed analogs showed that they could represent a feasible alternative to increase the market offer of these kinds of products.

### 3.4. Shelf-Life Study

**Figure 4:**
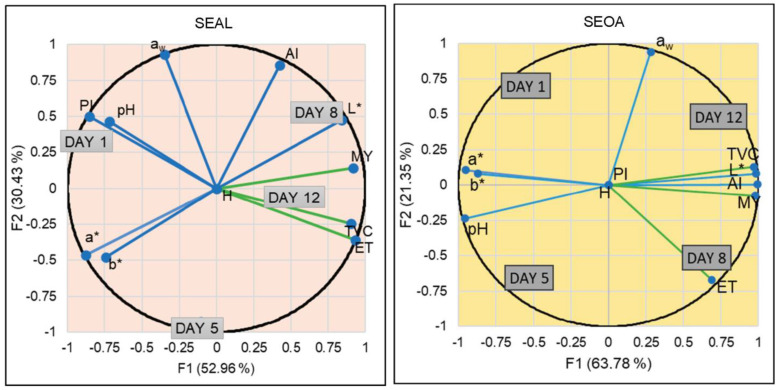

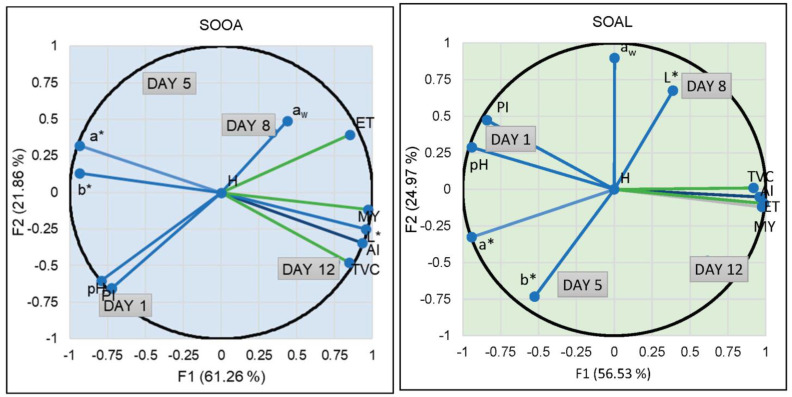
PCA of the shelf-life of the different formulations- a_w_: Water activity; AI: Acidity index; PI: Peroxide index; H: Moisture; L: Luminosity; a* and b*: Color coordinates; ET: *Enterobacteriaceae*; TVC: Total Viable Counts; MY: Molds and yeasts.

The shelf-life was studied through the following PCA graphics, where the physicochemical and microbiological parameters are plotted over time (Figure 4).

As can be seen in a general view, none of the PCAs showed a trend in linearity over time and all of them were different from each other. This means that the days follow one after the other in chronological order. In the SEAL sample, the variability of the experiment was found to be 83.39% for the two components. Day one was characterized by the physicochemical parameters of PI and pH. Over time, day five was distinguished by the color coordinates (a* and b*) experiencing a higher microbial load. Day 8 was described by AI and L* approached the final day (day 12) with the growth of microorganisms (TVC, MY, and especially ET, with a high ET contribution in the component). However, the sample formulated with seitan and oat (SEOA) behaved differently. The variability of the experiment was found to be 85.13% in the two components. The initial days were mainly characterized by color coordinates and pH. Over time, on day eight, microbial growth was again experienced until the end of its shelf-life, which was related to water activity. The physicochemical parameters such as acid value and lightness were described with the microbial growth, which is related to the higher content of free acids that determine the rancidity reactions. In this formulation, the peroxide value and moisture content were found to be neutral. The PI values were very low, which may be because some of the ingredients have antioxidant capacity. In the SOOA sample, the variability in the two components was 83.13% and again there was a relationship between the initial day with pH and PI. The color parameters (a* and b*) characterized day five, while day eight was defined by water activity, microbial growth, and AI until the end of shelf-life. For the SOAL sample, the variability of the experiment was described as 81.5% in the two components. Day one was again described by PI and pH, as is the case for the other alginate formulation, as shown in Table 2. Day five is described by the coordinates of more weight, a* and b*, in this case, due to a more yellowish coloration. Day eight was defined by water activity and luminescence. At the end of life, AI-related microbial growth began to occur.

#### Shelf-Life Study Model (Multivariate Method)

**Figure 5:**
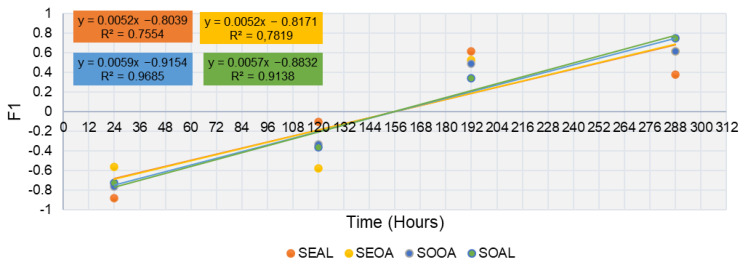
Coordinates in the first component according to times for the samples.

Figure 5 shows the shelf-life using the multivariate approach. It was estimated by plotting the scatter plots from the factorial scores of the first component (F1). These were obtained from the PCA graphics shown in Figure 4 based on the physicochemical and microbiological parameters evaluated. The intersection of the curve with the abscissa should be understood as the time that the product is kept in suitable conditions for consumption. These results estimated that the expiry date of all developed products was set at 156 h, i.e., 7 days. The samples showed no difference. This could be due to the similarities among formulations.

### 3.5. Spoilage Descriptive Model

**Figure 6:**
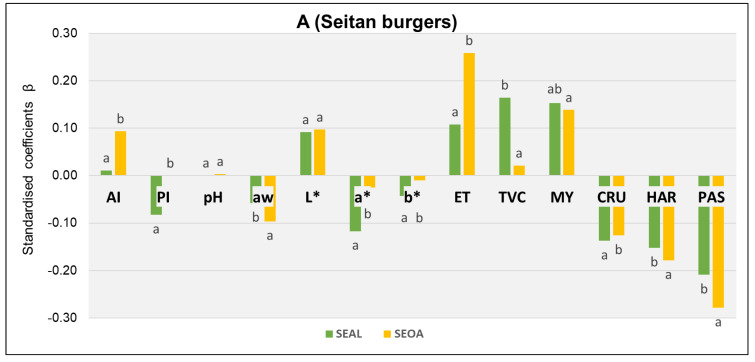

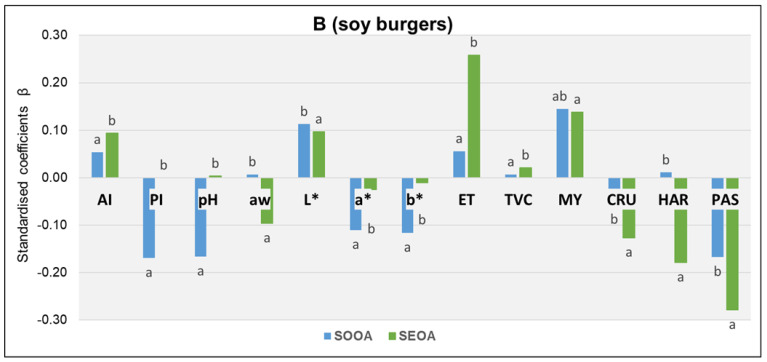
Standardized beta coefficient over time. L*: Luminosity; a* and b*: Color coordinates; ET: Enterobacteriaceae; TVC: Total Viable Counts; MY: Molds and yeasts; aw: Water activity; AI: Acidity index; PI: Peroxide index; H: Moisture; CRU: Crunchiness; HAR: Hardness; PAS: Pastiness. Different letters indicate significant differences (*p* < 0.05). (**A**): containing seitan (hamburgers for all the population except coeliacs), (**B**): containing soya (hamburgers suitable for coeliacs).

Partial least squares (PLS) regression was performed to model the multivariate data collected for the study to establish relationships between the parameters evaluated for the burger analogs. All studied variables (physicochemical, microbiological, and sensory freshness index) were considered to carry out a specific methodology indicated by Calanche et al. (2020) [38]. The PLS provided a time-dependent spoilage model as shown in Figure 6. In the initial time, a higher crunchiness was observed in the oat-based products. Over time, a change in attributes was experienced, with defects prevailing and loss of hardness being maximized. This may be due to the loss of water-holding capacity of the protein due to degradation causing bacterial proliferation.

A comparison of burger formulations could be made according to consumption, for all consumers and for the coeliac population. The treatments containing seitan in their formulation (for all consumers) showed a time-dependent relationship with microbial growth, in this case, influenced to a greater extent by the growth of enterobacteria. Physicochemical parameters such as acid index and brightness were the key aspects of this. Sensory texture attributes crunchiness, hardness, and pastiness were not directly related to spoilage. Significant differences were found between the binders chosen for the formulation, indicating that brightness was the only parameter constant over time in food spoilage. Degrading of the SEAL treatment was described by lightness (L*) and to a greater extent by the growth of aerobic mesophiles, molds, and yeasts. In treatments incorporating oat, spoilage was conditioned by the acidity index, brightness, and the growth of enterobacteria. In the case of formulations designed with soya, they showed high values of β coefficients that demonstrated a relevant impact on the deterioration model obtained. In this case, microbial growth was linked to the *Enterobacteriaceae* family and molds and yeasts. To a lesser extent, the sensory attribute of hardness was affected by storage time, indicative of spoilage in both formulations. Acid index values and water activity were the main factors indicating deterioration in the soybean formulations. pH and PI were not related to deterioration in these samples. The binders used showed differences between them. In the case of alginate, there was a very high AI value related to aw and growth of enterobacteria, molds, and yeasts. Whereas, those incorporating oats in their formulation stood out for brightness as a spoilage factor and the growth of molds and yeast.

Finally, the spoilage of the burger was mainly conditioned by the proliferation of microorganisms and was characterized by AI and luminosity (L*) on the surface of the samples. In terms of sensory attributes, most of the treatments developed did not show considerable changes; however, it could be seen that textural properties were a key aspect of this study.

## 4. Conclusions

The development of burger analogs from non-traditional raw materials of vegetable origin and enriched with insect meal is presented as a technologically feasible and nutritionally interesting option to offer alternative and innovative food products. The iterative and heuristic process allowed the development of four prototypes: two for all consumers and two specifically for coeliacs. There were differences in the physicochemical and sensory qualities of the different prototypes. The characterization of the initial product by quantitative descriptive analysis led to significant differences mainly in the texture dimension. Each formulation was characterized by different attributes, with oat-based formulations being closer to each other. The multivariate shelf-life study defined a “best before date” for all developed burger analogs in 156 h, i.e., 7 days, packed in an air tray, which was limited by bacterial growth. The spoilage model confirmed that microbial development together with texture parameter changes were the main cause of the loss of quality for new products manufactured. In summary, since the objectives have been met, further research on these new products is expected in the future.

## Figures and Tables

**Table 1:** Final formulations of burger analogs.

Ingredients	SEOA (%)	SOOA (%)	SEAL (%)	SOAL (%)
Texturized soya	0	62	0	73.8
Seitan	62	0	75	0
Insect flour	15	15	15	15
Water	2.7	5.3	0.5	0
Sodium Alginate	0	0	1.3	1.25
Calcium chloride	0	0	0.5	0.5
Oat flour	12.6	10	0	0
Vegetable fat	5	5	5	5
Salt	1.5	1.5	1.5	1.5
Beetroot powder	0.5	0.5	0.5	0.5
Flavoring	0.2	0.2	0.2	0.2
Sweet paprika powder	0.1	0.1	0.1	0.1
Smoke aroma	0.2	0.2	0.2	0.2
Aroma	0.2	0.2	0.2	0.2

SEOA: Burger analog seitan + oat; SOOA: Burger analog texturized soya + oat; SEAL: Burger analogue seitan + alginate; SOAL: Burger analogue texturized soya + alginate.

**Table 2:** Physicochemical parameters for burger analogs.

Samples	AI	PI	pH	a_w_	H	L*	a*	b*
SEAL	53.22 ^c^	1.736 ^d^	6.470	0.982	57.300	36.740 ^a^	8.730 ^a^	18.390
SOAL	41.182 ^b^	1.227 ^c^	6.420	0.978	52.420	44.295 ^c^	9.585 ^b^	19.725
SOOA	34.,861 ^a^	0.849 ^b^	6.410	0.979	58.810	43.245 ^b,c^	9.950 ^b^	16.440
SEOA	39.139 ^a,b^	0.008 ^a^	6.390	0.981	49.270	42.250 ^b^	8.900 ^a^	19.425
*Pr* > *F*	0.001	0.000	>0.05	>0.05	>0.05	0.002	0.016	>0.05

The values in this table correspond to the mean of three replicates (n = 3). AI: Acidity index (g Oleic Acid/100 g product); PI: Peroxide Index (meq O_2_/Kg); a_w_: Water activity; H: Moisture (%); L*: Luminosity; a*: Red/green coordinates; b*: Yellow/blue coordinates. SEOA: Burger analog seitan + oat; SOOA: Burger analog texturized soya + oat; SEAL: Burger analog seitan + alginate; SOAL: Burger analog texturized soya + alginate. Different letters in the same column denote statistically significant differences (*p* < 0.05) among samples for the same parameter.

## Data Availability

Data are contained within the article.
